# Enhanced sensitivity to cholera toxin in female ADP-ribosylarginine hydrolase (ARH1)-deficient mice

**DOI:** 10.1371/journal.pone.0207693

**Published:** 2018-11-30

**Authors:** Kizuku Watanabe, Jiro Kato, Jianfeng Zhu, Hirotake Oda, Hiroko Ishiwata-Endo, Joel Moss

**Affiliations:** 1 Pulmonary Branch, National Heart, Lung, and Blood Institute, National Institutes of Health, Bethesda, Maryland, United States of America; 2 Present address: Siemens Healthcare Diagnostics Inc., Newark, Delaware, United States of America; East Carolina University Brody School of Medicine, UNITED STATES

## Abstract

Cholera toxin, an 84-kDa multimeric protein and a major virulence factor of *Vibrio cholerae*, uses the ADP-ribosyltransferase activity of its A subunit to intoxicate host cells. ADP-ribosylation is a posttranslational modification of proteins, in which the ADP-ribose moiety of NAD^+^ is transferred to an acceptor. In mammalian cells, ADP-ribosylation of acceptors appears to be reversible. ADP-ribosyltransferases (ARTs) catalyze the modification of acceptor proteins, and ADP-ribose-acceptor hydrolases (ARHs) cleave the ADP-ribose-acceptor bond. ARH1 specifically cleaves the ADP-ribose-arginine bond. We previously demonstrated a role for endogenous ARH1 in regulating the extent of cholera toxin-mediated fluid and electrolyte abnormalities in a mouse model of intoxication. Murine ARH1-knockout (KO) cells and ARH1-KO mice exhibited increased sensitivity to cholera toxin compared to their wild-type (WT) counterparts. In the current report, we examined the sensitivity to cholera toxin of male and female ARH1-KO and WT mice. Intestinal loops derived from female ARH1-KO mice when injected with cholera toxin showed increased fluid accumulation compared to male ARH1-KO mice. WT mice did not show gender differences in fluid accumulation, ADP-ribosylarginine content, and ADP-ribosyl Gαs levels. Injection of 8-Bromo-cAMP into the intestinal loops also increased fluid accumulation, however, there was no significant difference between female and male mice or in WT and KO mice. Female ARH1-KO mice showed greater amounts of ADP-ribosylated Gαs protein and increased ADP-ribosylarginine content both in whole intestine and in epithelial cells than did male ARH1-KO mice. These results demonstrate that female ARH1-KO mice are more sensitive to cholera toxin than male mice. Loss of ARH1 confers gender sensitivity to the effects of cholera toxin but not of cyclic AMP. These observations may in part explain the finding noted in some clinical reports of enhanced symptoms of cholera and/or diarrhea in women than men.

## Introduction

*Vibrio cholerae* infection is a significant health problem in many parts of the world. According to the World Health Organization (WHO) annual report in 2016, 172,454 cases including 1,304 deaths were recorded in 42 countries [1]. Some areas still show high case fatality rates, probably because of inadequate case management or delays in initiating treatment [1].

Cholera toxin, an 84-kDa multimeric protein, is a major virulence factor of *Vibrio cholerae* [2, 3]. The toxin consists of a single enzymatically active A subunit (CTA) and five receptor-binding B subunits (CTB) [3]. The CTB forms a pentametric ring that is associated with CTA. Isolated CTA and CTB are nontoxic [2]. CTB has a strong affinity for its cell surface receptor, ganglioside GM1, and facilitates CTA endocytosis into cells [4]. CTA is an ADP-ribosyltransferase that catalyzes the modification of the α subunit of the Gs protein (Gαs), which is responsible for activation of adenylyl cyclase [5, 6]. ADP-ribosylation of Gαs stabilizes its active GTP-bound form [6]. Active ADP-ribosylated Gαs increases the activity of the catalytic unit of adenylyl cyclase, leading to accelerated cyclic AMP (cAMP) production [7]. As a consequence of the increased intracellular cAMP concentration, an imbalance in electrolyte transport occurs across the epithelial cell membrane [8]. Water flows in response to the ion gradient, resulting in watery diarrhea and fluid loss [9, 10].

ADP-ribosylation is a posttranslational modification of proteins, in which the ADP-ribose moiety of NAD is transferred to an acceptor (e.g., protein) [11]. Both mono- and poly- ADP-ribosylation have been recognized based on the number and method of attachment of ADP-ribose moieties to acceptors [11]. In poly-ADP-ribosylation, branching polymers of ADP-ribose, are attached to target amino acid residues (e.g. poly-ADP-ribose-polymerase (PARP)-1) [12]. Poly-ADP-ribosylation is induced in part by genomic stress and plays a role in chromosomal stability, regulation of transcription, DNA repair, telomere homeostasis, and oncogenesis [12]. In mono-ADP-ribosylation, a single ADP-ribose moiety of NAD^+^ is attached to an amino acid in a target protein [13]. Mono-ADP-ribosylation was first discovered as a mechanism used by bacterial toxins such as cholera toxin, diphtheria toxin, and pertussis toxin to disrupt biosynthetic and signal transduction pathways [5, 14–16]. Mammalian cells have endogenous ADP-ribosyltranferases (ARTs) that catalyze the ADP-ribosylation of acceptor proteins, reactions similar to those catalyzed by bacterial toxins [13, 17, 18]. In these cells, the extent of ADP-ribosylation is regulated in part by ADP-ribosyl-acceptor hydrolases (ARHs) that cleave the ADP-ribose-acceptor bond, regenerating the unmodified acceptor [19]. Thus, in mammalian cells, ADP-ribosylation appears to be a reversible modification of proteins [20, 21].

Five ADP-ribosyl transferase family members (ART1-5) have been cloned from mammalian cells with a related avian enzyme [13, 22]. ART1-4 are glycosylphosphatidylinositol (GPI)-anchored to cell membranes, whereas ART5 is a secreted protein. Mouse and human ART1 and ART5, and mouse ART2 transfer an ADP-ribose from NAD^+^ to arginine residues of acceptor proteins [22–24]. Substrates for ART3 and ART4 have not been identified [13, 24, 25]. In terms of ADP-ribose-acceptor hydrolases, three 39-kDa family members have been identified (ARH1-3) that share similar amino acid sequences [19]. Among the ARH family members, only ARH1 cleaves the ADP-ribose-arginine bond [26, 27].

In a prior study [28], we reported a role of ARH1 in a mouse model of intoxication of small intestinal epithelial cells and mouse embryonic fibroblasts by cholera toxin. CT increased ADP-ribose (arginine) protein content and ADP-ribosylated Gαs in murine ARH1-knockout (ARH1^-/-^, ARH1-KO) cells more than in their wild-type (WT) counterparts. In addition, ADP-ribose (arginine) protein content and ADP-ribosylated Gαs were significantly reduced by overexpression of wild-type ADP-ribosyl-acceptor hydrolase proteins in ARH1^-/-^ cells. We observed that, in response to CT treatment, ARH1-KO mice demonstrated a greater increase in fluid accumulation, Gαs modification, and ADP-ribosylarginine content in intestinal loops than their wild-type littermates. These data suggest that ADP-ribosylation is critical for cholera toxin action and that ARH1 plays an important role in controlling the intoxication process.

Some infectious diseases show gender bias in outcomes that may be explained by the Physiological Hypothesis (PH) and/or the Behavioral Hypothesis (BH) [29]. The PH postulates that sex hormones affect the immune system to modify susceptibility to disease. The PH also postulates a difference in immune status, resulting from the fact that some genes encoding immune-related proteins are located on the X chromosome [30]. Alternatively, the BH postulates that gender-specific or gender-biased behavior results in sex-biased infection rates, that is, gender differences in treatment outcome. Differences of infection rates are supposed to result from differences in societal norms such as domestic responsibility for caring for the sick, time spent at home, and access to health care [31]. There is still a controversy over whether gender differences exist in incidence and mortality of diarrheal diseases including cholera [32–38]. However, some reports claim that female sex is an individual risk factor for cholera incidence or death from diarrhea [35–38]. In this report, we focused on biological differences in reaction to CT between genders in WT and ARH1-KO mice.

## Materials and methods

### Materials

Cholera toxin was purchased from List Biological Laboratory, Inc, California, USA; 8-Bromo-cAMP and trichloroacetic acid (TCA) were purchased from Sigma-Aldrich Inc, St. Louis, USA; boronate resin (Affi-Gel boronate) was purchased from Bio-Rad Laboratories, Inc; 12% Tris-glycine or 12% bis-Tris gels were purchased from Invitrogen, California, USA; anti-rabbit polyclonal secondary antibodies were purchased from Promega Corporation, Wisconsin, USA; chemiluminescent substrate (Pierce SuperSignal West Pico and Femto) was purchased from Thermo Fisher Scientific Inc, Massachusetts, USA. Rabbit anti-Gαs polyclonal antibodies were provided by Dr. Lee Weinstein (NIDDK, NIH) and obtained from Sigma-Aldrich (St. Louis, MO) (C-terminal 385–394, #371732).

### Animal studies

Generation of ARH1-knockout mice was described previously [28]. Animal protocols (H-0127 and H-0172) were approved by the National Heart, Lung, and Blood Institute Animal Care and Use Committee. Knockout (ARH1^-/-^) and wild-type (ARH1^+/+^) mice were all littermates from heterozygous (ARH1^+/-^) breeding pairs. ARH1 mice were backcrossed 7 times using C57BL/6J mice. Mouse genotypes were confirmed by PCR using genomic DNA extracted from mouse tails using primers described in a previous report [28].

### Induction of fluid accumulation by cholera toxin (CT)

Fluid accumulation in mouse intestinal loops in response to CT was performed in ARH1^+/+^ and ARH1^-/-^ mice [28, 39]. After mice were anesthetized, intestine was exteriorized through a midline incision. Two or three intestinal segments of about 4 cm length each were generated by ligation with nylon suture, and 0.2 ml of PBS or PBS containing either 0.5 μg CT or 5 mM 8-Bromo-cAMP were injected into each loop [28]. Two to three drops of 0.5% bupivacaine were applied for analgesia after abdominal closure. Mice recovered from anesthesia on a warm-heated mat at 37°C. Following the study, mice were euthanized with carbon dioxide gas according to protocol [28]. Weight, length, and contents of each intestinal loop were recorded, and accumulated fluid was reported as weight per length (mg/cm) [28]. Data from six experiments were summarized as means ± standard errors of means (SEMs).

### ADP-ribosylarginine content

ADP-ribosylarginine content of mouse intestinal loops and luminal epithelial lining cells was quantified using samples that had been precipitated with 20% (w/v) TCA [28]. TCA-precipitated samples were centrifuged at 20,000 x g for 30 min, and supernatants were discarded; pellets were washed once with ice-cold TCA and twice with ether; residual ether was removed under vacuum, and samples were stored at -80°C. TCA-precipitated proteins (4 mg) were dissolved in 2 ml of 6 M guanidinium chloride containing 50 mM morpholinepropanesulfonic acid and 10 mM EDTA, pH 4.0, using a Dounce all-glass hand homogenizer (Kontes/Kimble Chase, TN, USA) with 30 strokes on ice. A 0.5 ml aliquot was incubated in a column of Affi-Gel boronate resin and was eluted with 5 ml of 0.1M glycine equilibrated at pH 9.0 containing 0.1M NaCl and 10 mM magnesium chloride to obtain fluorescent derivatives [40], which were analyzed by High-Performance Liquid Chromatography (Agilent Technologies Inc., California, USA). Samples were assayed in triplicate, and data are reported as means of values from 6 experiments.

### Western blot analysis

Collected intestinal loops or luminal epithelial lining cells from intestinal loops were excised and immediately immersed in liquid nitrogen at -80°C. After liquid nitrogen was evaporated, 2 ml of ice-cold 20% TCA were added to frozen tissues, which were homogenized on ice using an all-glass hand homogenizer with 30 strokes. After homogenization, samples (20 μg of total protein) were added to 4-fold SDS buffer and directly applied to SDS-PAGE in a 10% Tris-glycine gel (Thermo Fisher Scientific, MA) or 10% polyacrylamide gels (200 x 200 x 1.5 mm, Hoffer SE600). Proteins were resolved and transferred to nitrocellulose membranes (Thermo Fisher Scientific, MA), which were incubated with rabbit anti-Gαs polyclonal antibody at a ratio of 1:1000. After incubation with anti-rabbit polyclonal secondary antibody (Promega, WI) at a ratio of 1:2500, the protein bands were visualized with Chemiluminescent Substrate (Thermo Fisher Scientific, MA) and visualized with X-ray film (Kodak, New York, USA). Blotting images were scanned by Imaging scanner.

### Pull-down of ADP-ribosylated Gαs using Af1521 macro-domain-GST

To detect ADP-ribosylation of Gαs following intestinal loop treatment with PBS or cholera toxin, mouse intestinal loops without fluid were immediately frozen in liquid nitrogen and ground in a mortar. After evaporation of liquid nitrogen, 8% (w/v) ice-cold TCA was added. TCA-precipitated proteins were dissolved in 4 ml of ice-cold Tris-HCl buffer (20 mM Tris-HCl, pH 7.5, 20 mM NaCl). Protein was quantified by spectrophotometer using Pierce BCA Protein Assay Kit (Thermo Fisher Scientific, MA).

Af1521 macro-domain-GST (0.5 μmol/100 μl) or inactive Af1521 macro-domain-GST (0.5 μmol/100 μl) was pre-incubated for 1 hour at 4°C with β-NAD (1 μmol/100 μl), ADP-ribose (1 μmol/100 μl) or PBS, and then incubated with TCA-treated mouse intestinal loops (100 μg/reaction) overnight at 4°C in a pull-down assay to concentrate ADP-ribosylated Gαs, which then was identified by immunoreactivity. Also, the dissolved TCA-treated mouse intestinal loop lysates in Tris-HCl buffer (100 μg/100 μl) with 5 mM MgCl_2_ were incubated with mouse recombinant ARH1 proteins (0.5 μg/10 μl) for 1.5 hour at 37°C, and then incubated with Af1521 macro-domain-GST (0.5 μmol/100 μl) overnight at 4°C in a pull-down assay.

For pull-down of ADP-ribosylated Gαs with Af1521 macro-domain-GST resin (Tulip BioLabs, Lansdale, PA), intestine lysates were incubated with 20 μg of Af1521 macro-domain-GST resin with a rotator (20 reversals/min) overnight at 4°C and washed three times. Proteins were separated by SDS-PAGE using 4–12% bis-Tris NuPAGE gels (Thermo Fisher Scientific, MA) and transferred to nitrocellulose membranes. Blotted membranes were blocked with TBS-T (Tris-buffered saline, 0.1% Tween 20) with 5% skim milk (Bio-Rad) at room temperature (RT) for 1 h, then reacted with rabbit anti-Gαs antibody 1:1000 dilution (C-terminal 385–394, Sigma, St. Louis, MO) overnight at 4°C. After washing with TBS-T at RT for 10 min, 3 times, membranes were reacted with HRP-conjugated, anti-rabbit IgG (1:2500 dilution, Promega) at RT for 1 h. Washing was performed as above. Enhanced chemiluminescence substrate, Pierce SuperSignal West Femto (Thermo Fisher Scientific, MA) was used for visualization of immunocomplexes, which were detected with Fujifilm LAS-4000 (Fujifilm).

### Statistics

Data were analyzed with a Student’s t-test and presented as means ± SEMs, with a *P*-value of <0.05 considered to be significant.

## Results

### ADP-ribosylated Gαs is an ARH1 substrate

**Fig 1:**
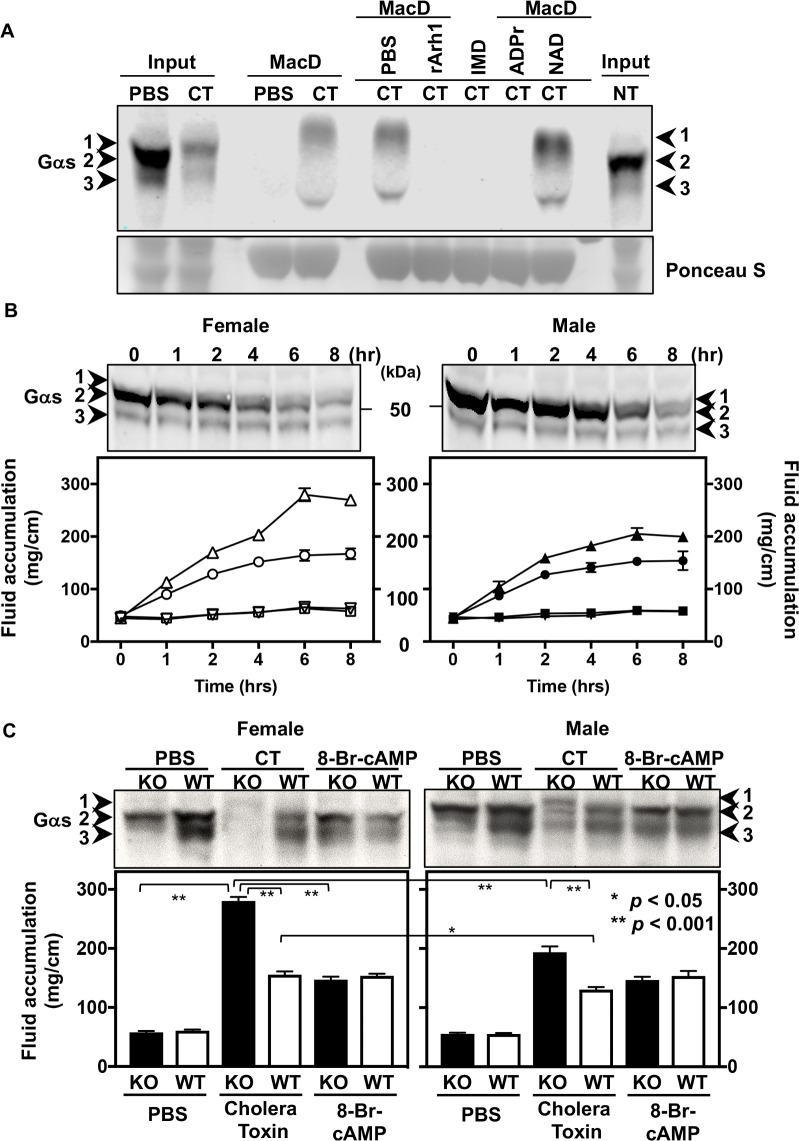
Effects of ARH1 genotype and gender on fluid accumulation stimulated by cholera toxin (CT) in intestinal loops. (A) Immunoblots using Gαs antibody show the ADP-ribosylated Gαs in ARH1 KO intestinal loops treated with PBS or cholera toxin (CT) for 6 hours quantified by active (MacD) or inactive (IMD) Af1521 macro-domain GST pull-down assay that was pre-treated with PBS, 0.1 mM ADP-ribose (ADPr) or 0.1 mM β-NAD (NAD) for 1 hr at 4°C. Recombinant ARH1 proteins (rArh1) (0.5 μg/10 μl) were incubated with TCA-treated intestinal loop lysates of CT-treated loops for 1.5 hrs at 37°C before assaying with Af1521 macro-domain GST pull-down. Ponceau S staining of same blot membranes shows the amount of Af1521 macro-domain using GST pull-down assay. These data were repeated in duplicate and in four experiments (n = 4) using TCA-precipitated ARH1 KO intestinal loops after exposure for 6 hours to PBS, CT or non-treatment (NT) (Input 40 μg/lane, 100 μg protein was assayed with Af1521 pull-down). (B) Above blots show Gαs in ARH1 KO female and male intestinal loops after exposure to cholera toxin (0.5 μg/0.2 ml) for 0 to 8 hours (hr) as indicated. Numbered arrowheads are indicated as follows: 1, ADP-ribosylated Gαs; 2, 52-kDa Gαs; and 3, 45-kDa Gαs band. Time course blot data were repeated in duplicate and in three experiments (n = 3) using TCA-precipitated ARH1 KO intestinal loops treated with cholera toxin (CT) (40 μg/lane). Intestinal loops in each WT female (▽-PBS or ○-CT), KO female (□-PBS or △-CT), WT male (▼-PBS or ●-CT) and KO male (■-PBS or ▲-CT) were injected with PBS (0.2 ml) and cholera toxin (0.5 μg/0.2 ml) separately and were measured at the indicated time after injection. Fluid accumulation were measured using the weight (mg) and length (cm) in each loop at indicated time. Data are means ± SEM of values for fluid accumulation (n = 6). Effects of genotype (p < 0.001), gender (p < 0.001), treatment (p < 0.001) or interaction between time and gender (p < 0.001) were significant for fluid accumulation (2-way ANOVA, Tukey’s multiple comparison test). Pairwise comparisons were significantly different (p < 0.001) in KO female CT vs. KO male CT, KO female CT vs. WT female CT, KO male CT vs. WT male CT, but not significant in KO female PBS vs. KO male PBS, KO female PBS vs. WT female PBS, KO male PBS vs. WT male PBS. (C) Upper immunoblots using Gαs antibody shows the modified Gαs in epithelial cell from intestinal loop treated with PBS, cholera toxin (CT) or 8-Bromo-cAMP (8-Br-cAMP) in female KO and WT, and male KO and WT mice. Numbered arrowheads are indicated as follows: 1, ADP-ribosylated Gαs; 2, 52-kDa Gαs; and 3, 45-kDa Gαs band. Lower figure shows effects of ARH1 genotype and gender on fluid accumulation by cholera toxin in intestinal loops of ARH1 WT and KO mice. Intestinal fluid accumulation (mg/cm) in female or male ARH1 WT and KO mice was determined after exposure for 6 hours to PBS, or PBS containing 0.5 μg cholera toxin (CT) or 5 mM 8-Br-cAMP. Data are means ± SEM of values for fluid accumulation (mg/cm) from ten intestinal loops of ten mice in each treatment (n = 10). Effects of genotype (p < 0.001), gender (p < 0.001) or treatment (p < 0.001) are significantly different for fluid accumulation (2-way ANOVA, Tukey’s multiple comparison test). Pairwise comparisons were significant (p < 0.001) in KO female CT vs. KO male CT, and WT female CT vs WT male (p = 0.012), but not significant in KO female 8-Br-cAMP vs KO male 8-Br-cAMP (p > 0.9999).

Cholera toxin A subunit is an ADP-ribosyltransferase that catalyzes the modification of the α subunit of the Gs protein (Gαs), which is responsible for activation of adenylyl cyclase [5, 6]. To determine Gαs ADP-ribosylated by cholera toxin, Af1521, an ADP-ribose-binding macro domain, was used in a pull-down assay with TCA-precipitated samples from CT-treated intestinal loops in ARH1 KO mice. As expected, ADP-ribosylated Gαs from CT-treated intestinal loops was bound by Af1521. In samples treated with recombinant ARH1 protein, modified Gαs was not detected with Af1521. Further, ADP-ribosylated Gαs was bound by Af1521 in the presence of β-NAD. However, as expected, binding was blocked by free ADP-ribose. The inactive Af1521 macro domain mutant also did not bind ADP-ribosylated Gαs (Fig 1A and S1 Fig). On the same blot, a 52-kDa Gαs band from CT-treated intestinal loops was shifted up and showed less electrophoretic mobility compared with the bands from PBS-treated loops (Fig 1A and S1 Fig). Next, recombinant ARH1 hydrolyzed the ADP-ribose-arginine-Gαs products of the CT-catalyzed reaction. These data suggested that Gαs bands from CT-treated intestinal loops in ARH1 KO mice represent ADP-ribosylated Gαs.

### Effects of ARH1 genotype and gender on fluid accumulation by cholera toxin in intestinal loops

We quantified fluid accumulation in the mouse intestinal loop model and ADP-ribosylation of Gαs proteins in response to cholera toxin (CT). Intestinal loops in each WT female, KO female, WT male, and KO male mice were injected with PBS or cholera toxin, and fluid accumulation was measured every 2 hours (hr) at the indicated time. Accumulated fluid in each loop was recorded as weight (mg) and longitudinal length (cm). Quantified of CT treated fluid accumulation was clearly increased with time in both ARH1 KO female and male (Fig 1B and S2 Fig). Differences in volume of fluid accumulation were significant between CT-treated groups and PBS-treated groups of either gender and of either genotype. Pairwise comparisons showed significant differences between ARH1-KO females treated with CT (KO female CT) and ARH1-KO males treated with CT (KO male CT), KO female CT and WT female CT, KO male CT and WT male CT. No significant differences, however, were observed among PBS-treated groups between KO female PBS and KO male PBS, KO female PBS and WT female PBS, and KO male PBS and WT male PBS mice (Fig 1B and S2 Fig). Also, differences in the behavior of Gαs from intestinal loops of female and male ARH1 KO mice after CT treatment were significant (Fig 1B, upper blots and S2 Fig). Mobility of modified Gαs from female KO is less than that from male KO mice (Fig 1B, upper blots and S2 Fig). Modification of Gαs correlated with the volume of fluid accumulation in intestinal loops. These data suggested that intestinal loops from female ARH1 KO mice were more sensitive to modification of Gαs and fluid accumulation than those from male KO mice.

ADP-ribosylated Gαs proteins were extracted from epithelial cells from intestinal loops of ARH1-KO and WT mice of both genders that were treated for 6 hrs with PBS, CT, or 8-Bromo-cAMP, and were quantified by Western blot (Fig 1C, upper blots and S3 Fig). The cells of intestinal loops contained both 52-kDa and 45-kDa forms of Gαs. CT-catalyzed ADP-ribosylation decreased the mobility of Gαs in ARH1-KO cells. Time course data of CT-exposure from female ARH1 KO intestinal epithelial cells after 1 h of CT exposure and from male ARH1 KO epithelial cells after 4 h of CT exposure, where the amount of 52-kDa Gαs was reduced, are shown in Fig 1B, upper blot. Continued accumulation of ADP-ribosylated Gαs was not seen because the modified proteins were degraded as reported previously [28]. The loss of Gαs following CT exposure of epithelial cells in intestinal loops was dependent on time of CT exposure.

As shown in Fig 1C upper blots, the 52-kDa and 45-kDa Gαs protein bands in epithelial cells of intestinal loops in female ARH1-KO mice were diminished greatly in intensity while ADP-ribosylated Gαs appeared on the blot, whereas, in male ARH1-KO mice, 52-kDa and 45-kDa Gαs protein bands were still visible while ADP-ribosylated Gαs appeared. These data also suggested that the loss of 52-kDa and 45-kDa Gαs bands probably resulted from their ADP-ribosylation and shift in mobility.

As shown of the quantified of CT treated fluid accumulation in Fig 1B and 1C, the effects of genotype and gender on fluid accumulation were investigated. Accumulated fluid in the intestinal loops was quantified in ARH1-KO and WT mice of both genders after they were exposed for 6 hr to PBS, or PBS containing either 0.5 μg CT or 5mM 8-Bromo-cAMP. Differences in volume of fluid accumulation were significant when compared between genotype, gender, or treatment. Significant differences were observed between KO female CT-treated and KO male CT-treated, and between WT female CT-treated and WT male CT-treated mice, whereas no significant differences were observed between KO female 8-Bromo-cAMP and KO male 8-Bromo-cAMP (Fig 1C and S3 Fig). These results showed that cholera toxin enhanced fluid accumulation (6 hours) in intestinal loops of ARH1 KO mice more than in loops from WT mice. Also, CT-treated fluid accumulation in female ARH1 KO and WT mice was greater than that from male ARH1 KO and WT mice, respectively. However, the effects of 8-Bromo-cAMP on fluid accumulation were similar in WT and KO, female and male mice (Fig 1C and S3 Fig). As is known, CT increased adenylyl cyclase activity, leading to an increase in cellular cAMP through ADP-ribosylation and activation of Gαs. cAMP affects on water and electrolyte transport were shown in previous reports [8, 28]. These data suggested that responsiveness to the downstream signaling pathway was unchanged. Thus, the effects of cAMP on fluid accumulation in ARH1 KO and WT mice were independent of ARH1 activity.

### Effects of ARH1 genotype and gender on ADP-ribosylarginine content

**Fig 2:**
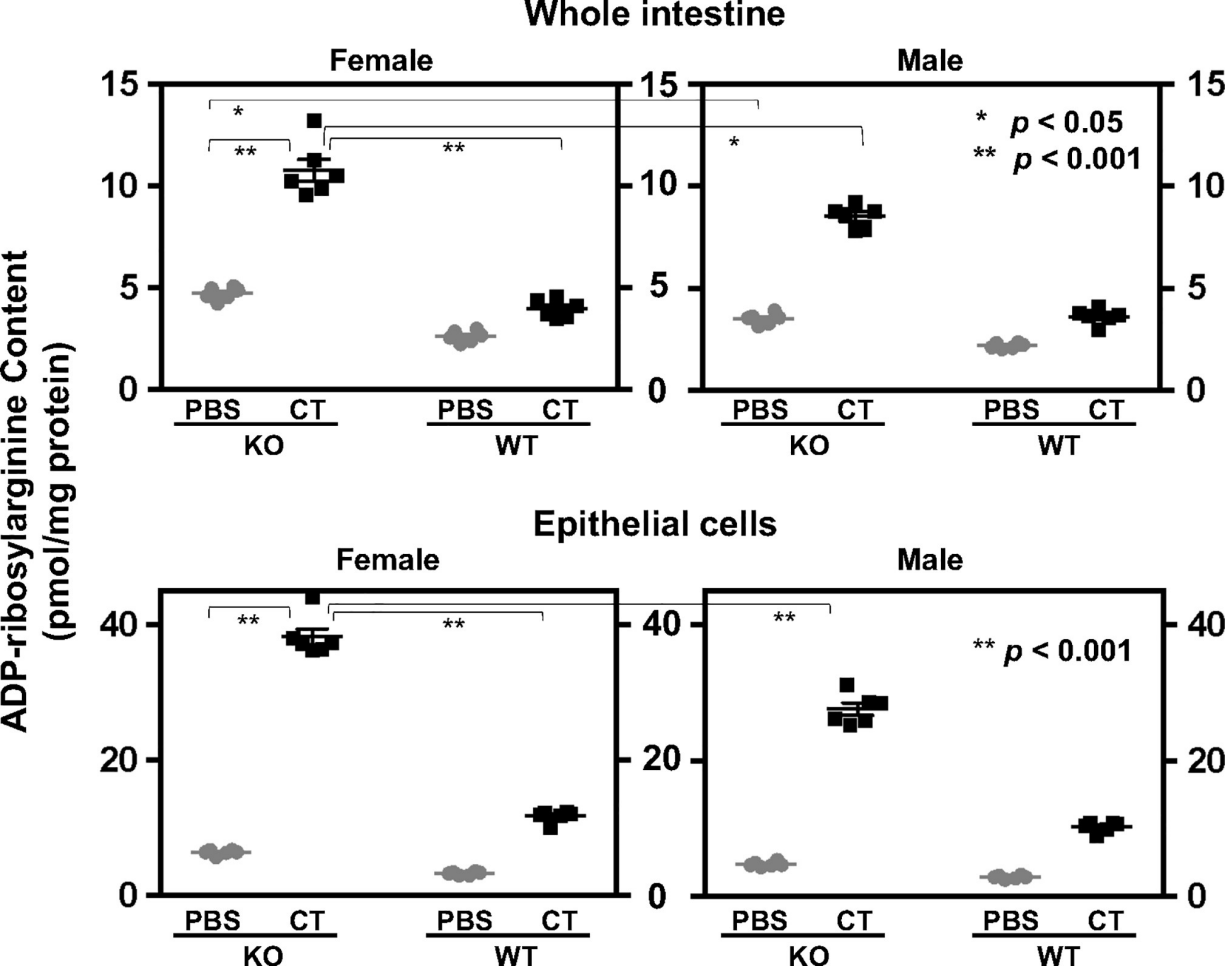
Effect of ARH1 genotype and gender on ADP-ribosylarginine content. ADP-ribosylarginine content of proteins from whole intestine (upper) or epithelial cells (lower) of loops were incubated with PBS or 0.5 μg of CT for 6 h. Data are means ± SEM of values (n = 6) from two loops of two mice in each of three experiments. Effects of genotype (p < 0.001), gender (p < 0.001) or treatment (p < 0.001) are significant for ADP-ribosylarginine content of proteins (2-way ANOVA, Tukey’s multiple comparison test). In whole intestine, pairwise comparisons were significantly different in KO male or female CT vs. KO male or female PBS (p < 0.001), KO female CT vs. KO male CT (p = 0.001) or KO female PBS vs KO male PBS (p = 0.0393), but differences were not significant (ns) for WT female CT vs. WT male CT (p = 0.9625) or WT female PBS vs. WT male PBS mice (p = 0.8972). In epithelial cells, differences were significant in KO male or female CT vs. KO male or female PBS (p < 0.001), KO female CT vs. KO male CT (p < 0.001), but not significant (ns) in WT female CT vs. WT male CT (p = 0.5815) or WT female PBS vs. WT male PBS mice (p = 0.9996).

Next, we measured ADP-ribosylarginine content in whole intestine as well as in epithelial cells. ADP-ribosylarginine content of proteins from whole intestine and intestinal epithelial cells was measured after the tissue was incubated with PBS or 0.5 μg of CT for 6 hrs. Cholera toxin appears to affect the epithelial cells on the inner surface of intestine, because epithelial cells contain gangliosides GM1, which binds cholera toxin and then promotes the ability of cholera toxin to ADP-ribosylate Gαs. Thus, the inner layer of cells was removed and analyzed separately from the remaining intestinal tissues. Differences between ADP-ribosylarginine content of proteins were significant when comparisons were made among genotype, gender, or treatment with or without CT. In whole intestine, pairwise comparisons showed significant differences between KO female (or male) CT-treated and KO female (or male) PBS-treated, KO female CT-treated and KO male CT-treated, and KO female PBS-treated and KO male PBS-treated. No significant differences were observed between WT female CT-treated and WT male CT-treated, and WT female PBS-treated and WT male PBS-treated. In epithelial cells, differences were significant between KO female (or male) CT-treated and KO female (or male) PBS-treated, and KO female CT-treated and KO male CT-treated; no significant differences were observed between WT female CT-treated and WT male CT-treated, and WT female PBS-treated and WT male PBS-treated (Fig 2). These results suggested that the ADP-ribosylated Gαs with CT-treated intestinal loops in ARH1 KO mice had more ADP-ribose(arginine)proteins compared with those in ARH1 WT mice. In summary, female ARH1-KO mice showed increased ADP-ribosylation of Gαs proteins and increased ADP-ribosylarginine content both in whole intestine and in intestinal epithelial cells than male ARH1-KO mice, consistent with the greater amounts of fluid accumulation in small intestine as well as greater extent of ADP-ribosylated Gαs.

## Discussion

In a prior study, we demonstrated a critical role of endogenous ARH1 in controlling CT activation in a mouse model of intoxication of small intestinal epithelial cells [28]. ARH1- knockout (ARH1^-/-^, ARH1-KO) cells showed increased ADP-ribose (arginine) protein content and ADP-ribosylated Gαs more than their wild-type (WT) counterparts when they were stimulated by CT [28]. In addition, the ADP-ribosylarginine content and ADP-ribosylated Gαs were significantly reduced by overexpression of wild-type ADP-ribosylarginine hydrolase proteins in ARH1^-/-^ cells. ARH1-KO mice demonstrated greater increase in fluid accumulation, Gαs modification, and ADP-ribosylarginine content in intestinal loops than their wild-type littermates in response to CT treatment. In this study, female ARH1-KO mice showed increased fluid accumulation in small intestine in response to cholera toxin than did male ARH1-KO mice. Further, female ARH1-KO mice, compared to their male counterparts, showed increased ADP-ribosylation of Gαs protein and greater ADP-ribosylarginine content both in whole intestine and in epithelial cells. Of note, the response to cAMP, the second messenger whose synthesis is stimulated by cholera toxin, did not differ between female and male ARH1-KO mice. The results of this study appear to indicate that ARH1-KO females are more prone to fluid loss resulting from watery diarrhea following intoxication by cholera toxin than ARH1-KO males.

Our report demonstrated gender differences of outcomes caused by CT intoxication. In agreement, recent meta-analysis revealed that women had a higher prevalence of cholera than men [41]; in addition, some reports claim that female sex is an individual risk factor for cholera incidence or death from diarrhea [35–38].

Intestinal peristalsis might be a potent cause of gender difference in fluid accumulation. A prior report revealed that CT on an isolated colon surface in C57BL/6 mice affected the neural regulation of contractile movement via 5-HT_3_ receptor-dependent pathway [42]. Female mice in estrus but not male mice or female mice in proestrus demonstrated reduced colon motility by CT. These effects resulted from a change of the number of 5-HT containing cells with the estrus cycle in female mice. Knockout of ARH1 might affect the status of 5-HT containing cells in small intestine, resulting in the gender differences, however, it has not been proven yet. Further, the intestine motility is limited by ligation of small intestine segments in small intestine loop models, and might not have affected the results of this study.

Other factors that might cause a gender difference by influencing innate host defense systems include gut microbiome [43]. Gut microbiome consists of symbiotic microbes, which is diverse among individuals and host-specific [44]. A prior study revealed that microbiota, which is recognized by CD11c-positive phagocytes via nucleotide-binding oligomerization domain containing 2 (Nod2), is a prerequisite for the antigen-specific IgG production enhanced by CT [45]. Some studies show that the gut microbiota show sex-specific differences in immunity in rodent models [46, 47]. It might be possible that ARH1-KO mice have an altered gut microbiome, which could have caused the sex bias in this study.

Surface proteins of the *Vibrio cholerae* bacterium and cholera toxin are the main antigens involved in mucosal immune reactions in small intestine [43]. Oral cholera vaccines are being developed to strengthen the adaptive immunity against inactivated bacterial surface protein and against cholera toxin B subunit, thus being protective against bacteria and cholera toxin. Interestingly, in clinical settings, oral cholera vaccine shows a protective effect in women more than men [48]. This result suggests that gender differences affect intestinal mucosal immunity. Several studies have shown that immune cells are activated and present in intestinal mucosa in diseased bowel [49, 50]. Nevertheless, the number of immune cells in the mucosa appear to be influenced by host gender or sex hormones in intestinal diseases [50–53].

Since cholera toxin was directly administered into murine small intestine, the results of our study do not necessarily reflect overall characteristics of *Vibrio cholerae* infection. Multiple defense mechanisms work in the host against *Vibrio cholerae* infection, keeping many people asymptomatic even when they are infected by toxin-producing cholera. *Vibrio cholerae* is transmitted by drinking pathogen-containing water or is highly likely transmitted from human to human through the fecal-oral route in endemic areas [54–56]. Most of the bacteria, however, appear to be destroyed by gastric acid [2, 57, 58]. In addition, cholera toxin does not activate adenylyl cyclase unless its A subunit is endocytosed into the epithelial cells [59]. To date, there seems no report addressing the gender differences in terms of these innate immune mechanisms.

Among over 200 types in *Vibrio cholerae* serotypes, cholera endemics are caused by either O1 or O139 serotypes. Further, O1 serotype is classified into the classical and the El Tor biotypes based on the biological factors and/or genetic markers of possessed toxin [60, 61]. It has been demonstrated that immunological responses seem to last longer in individuals with a previous history of classical biotype cholera than in those with a prior history of El Tor biotype cholera [62]. Thus, the response to *Vibrio cholerae* differs from person to person in the real world. It remains uncertain what proportion of men and women have previously acquired immunity to cholera based on prior exposure in endemic areas. The recurrent rates of cholera, particularly both in adult men and women, are affected individually by the state of immunity. However, in many societies, women seem to suffer from cholera more frequently than men [35–38, 41]. Societal norms such as caring for the sick or time to spend at home have been used to explain the exposure to cholera [31, 41]. The results of our study, however, appear to indicate that biological aspects may be responsible for some gender differences and outcomes in cholera epidemics. An underlying assumption in comparing WT and ARH1-KO mice is the possibility that the genetics of ARH1 differ between populations. Further investigation is needed as to whether individuals who suffer from recurrent cholera infections, particularly women, have dysfunctional regulation of ADP-ribose metabolism.

In summary, we reported biological differences in reaction to CT between male and female ARH1-KO mice. Based on the ARH1-KO and WT differences, it is possible that the level of ARH1 protein may affect the gender specificity of the response to cholera toxin. Expression and enzymatic function of ARH1 may be determined at the molecular levels by genetic factors such as polymorphisms. Considering that *Vibrio cholerae* infection is a significant health problem in many parts of the world, the results of this study may in part explain the reports of increased symptomatology of cholera toxin in women than in men.

## Supporting information

S1 Fig Effects of cholera toxin (CT) on ADP-ribosylation of Gαs in intestinal loops of ARH1 KO mice. S1 Fig presents the original Western blot (raw data) using rabbit anti-Gαs antibody and shows the ADP-ribosylated Gαs from ARH1 KO intestinal loops treated with PBS or cholera toxin (CT) for 6 hours. Lanes 2–14 in the original blot were shown in Fig 1A Gαs blot. Lanes 1, 4, 7 and 13 show the protein molecular weight (kDa) markers (PageRuler Plus Prestained Protein ladder, Thermofisher Scientific, MA).
(TIF)

S2 Fig Effects of cholera toxin (CT) on ADP-ribosylation of Gαs in intestinal loops of ARH1 KO female and male mice. S2 Fig presents the original Western blot (raw data) of Fig 1B Gαs blots. Left (female) and right (male) immunoblots using rabbit anti-Gαs antibody show Gαs from intestinal loops of ARH1 KO mice after exposure to cholera toxin (0.5 μg/0.2 ml) for 0 to 8 hours (hr) as indicated. Lanes 2–7 in both blots are shown in Fig 1B Gαs blots. Lane 1 shows the protein molecular weight (kDa) markers (PageRuler Plus Prestained Protein ladder, Thermofisher Scientific, MA).
(TIF)

S3 Fig Effect of ARH1 genotype and gender on modified Gαs in intestinal loop treated with PBS, cholera toxin (CT) or 8-Bromo-cAMP (8-Br-cAMP). S3 Fig presents the original blots (raw data) of Fig 1C Gαs blots.
Upper and Lower immunoblots using Gαs antibody show modified Gαs in intestinal loops treated with PBS, cholera toxin (CT) or 8-Bromo-cAMP (8-Br-cAMP) in female ARH1 KO and WT mice, and male ARH1 KO and WT mice, respectively. Lanes 2–7 in Western blots using anti-Gαs antibody was shown in Fig 1C.
Lanes 1 and 10 show the protein molecular weight (kDa) using See Blue Plus Protein marker (Invitrogen, CA). Lane 11: positive control, recombinant Gαs protein (50 ng) (Millipore Sigma, MA). Lanes 2–7; Intestinal loops in female or male ARH1 WT and KO mice were treated with PBS, PBS containing 0.5 μg cholera toxin (CT) or 5 mM 8-Br-cAMP for 6 hours. Lane 2: ARH1 KO intestinal loops treated with PBS, Lane 3: ARH1 WT intestinal loops treated with PBS, Lane 4: ARH1 KO intestinal loops treated with CT, Lane 5: ARH1 WT intestinal loops treated with CT, Lane 6: ARH1 KO intestinal loops treated with 5 mM 8-Br-cAMP, Lane 7: ARH1 WT intestinal loops treated with 5 mM 8-Br-cAMP.
In upper blot of female ARH1 KO mice; Lane 8: CT-treated intestinal loops for 2 hours in ARH1 KO mice, Lane 9: CT-treated intestinal loops for 4 hours in ARH1 KO.
In lower blot of male ARH1 KO mice; Lane 8: CT-treated intestinal loops for 4 hours in ARH1 KO mice, Lane 9: CT-treated intestinal loops for 2 hours in ARH1 KO.
(TIF)

S1 File NC3Rs ARRIVE guidelines checklist. (PDF)
