# Supplementary figures and images for: Enhanced sensitivity to cholera toxin in female ADP-ribosylarginine hydrolase (ARH1)-deficient mice
Source: PLoS One. 2018 Nov 30;13(11):e0207693. doi: 10.1371/journal.pone.0207693 (PMC6267974; doi:10.1371/journal.pone.0207693)

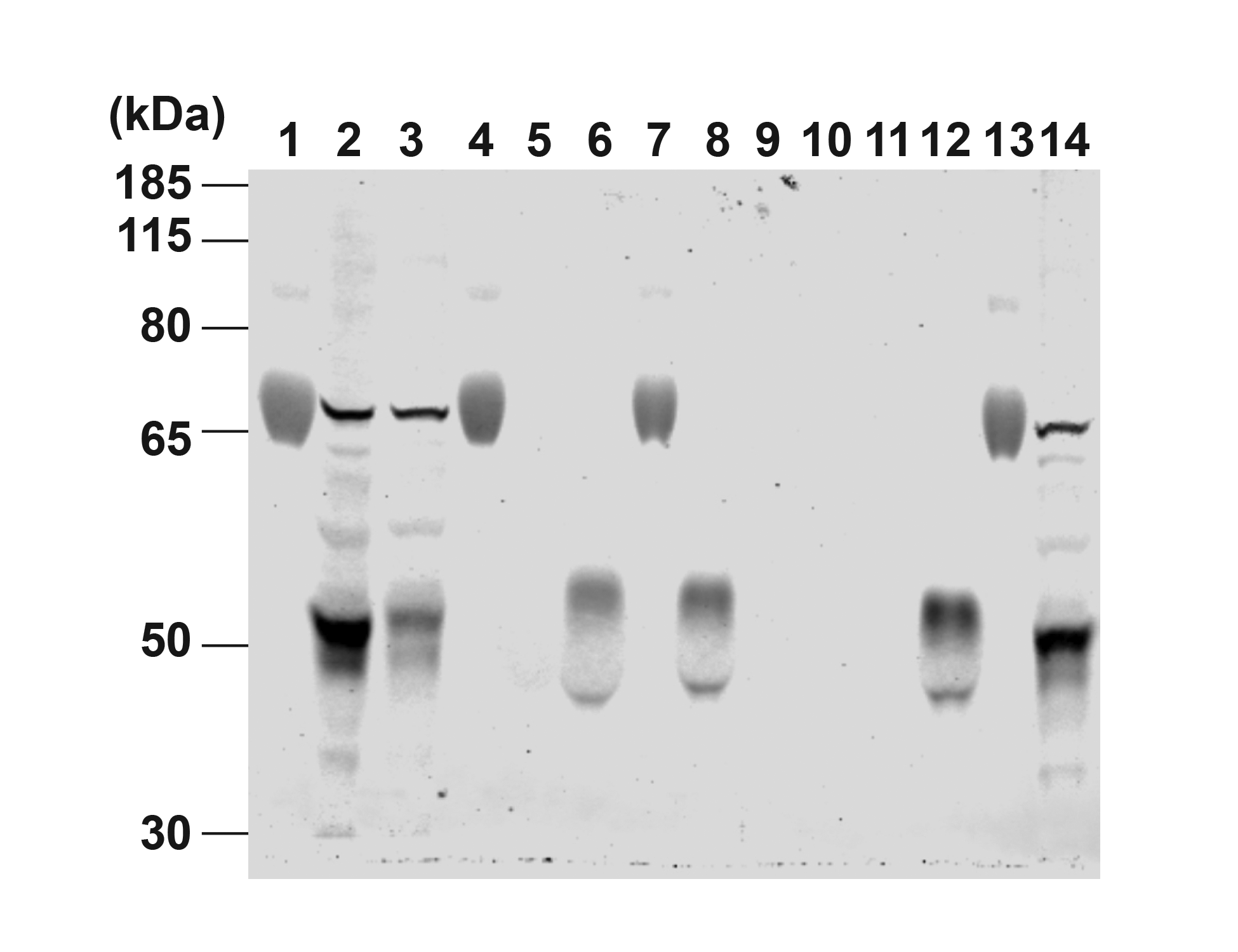

Supplement: S1 Fig — presents the original Western blot (raw data) using rabbit anti-Gαs antibody and shows the ADP-ribosylated Gαs from ARH1 KO intestinal loops treated with PBS or cholera toxin (CT) for 6 hours. Lanes 2–14 in the original blot were shown in Fig 1A Gαs blot. Lanes 1, 4, 7 and 13 show the protein molecular weight (kDa) markers (PageRuler Plus Prestained Protein ladder, Thermofisher Scientific, MA). (TIF) [file pone.0207693.s001.tif]

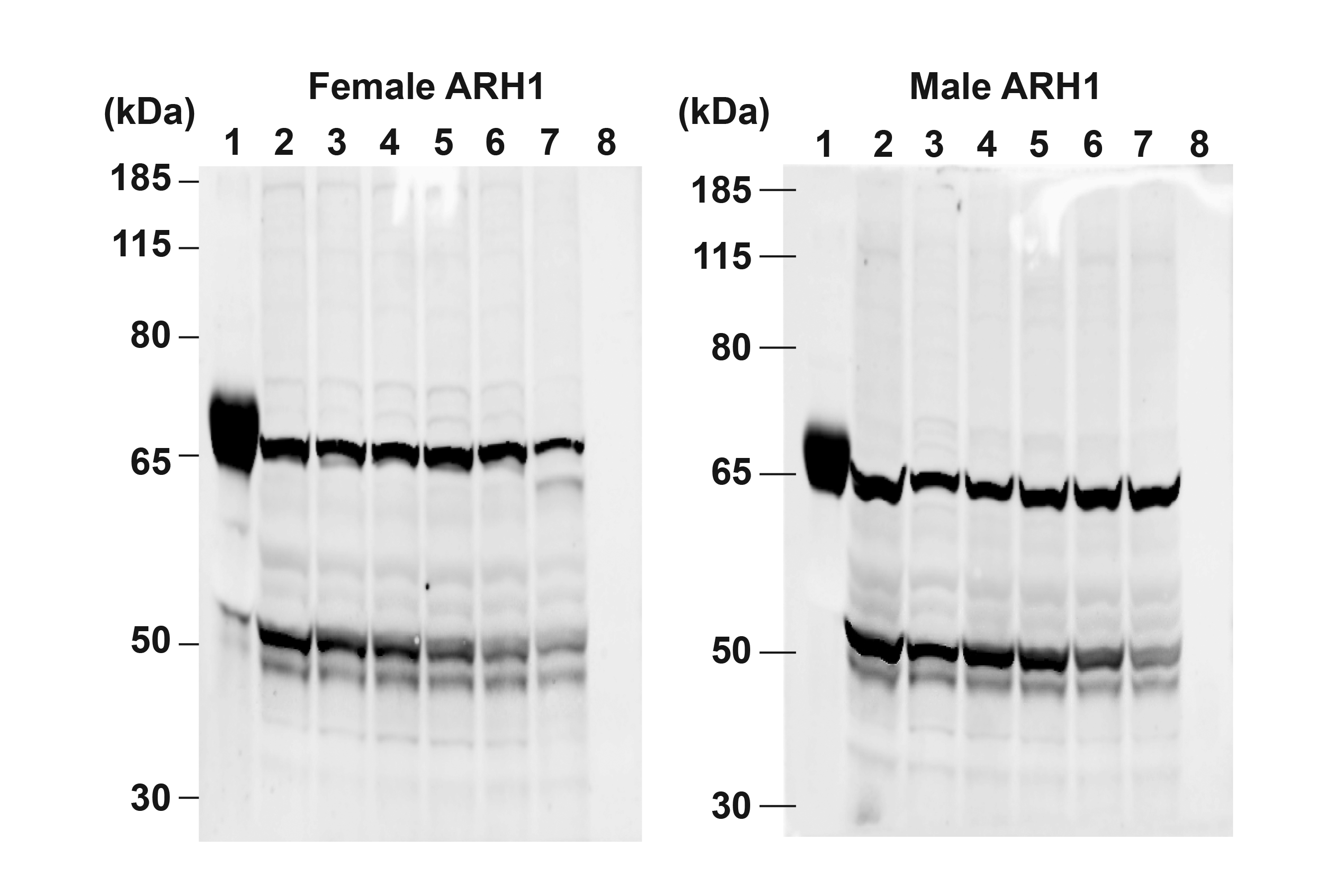

Supplement: S2 Fig — presents the original Western blot (raw data) of Fig 1B Gαs blots. Left (female) and right (male) immunoblots using rabbit anti-Gαs antibody show Gαs from intestinal loops of ARH1 KO mice after exposure to cholera toxin (0.5 μg/0.2 ml) for 0 to 8 hours (hr) as indicated. Lanes 2–7 in both blots are shown in Fig 1B Gαs blots. Lane 1 shows the protein molecular weight (kDa) markers (PageRuler Plus Prestained Protein ladder, Thermofisher Scientific, MA). (TIF) [file pone.0207693.s002.tif]

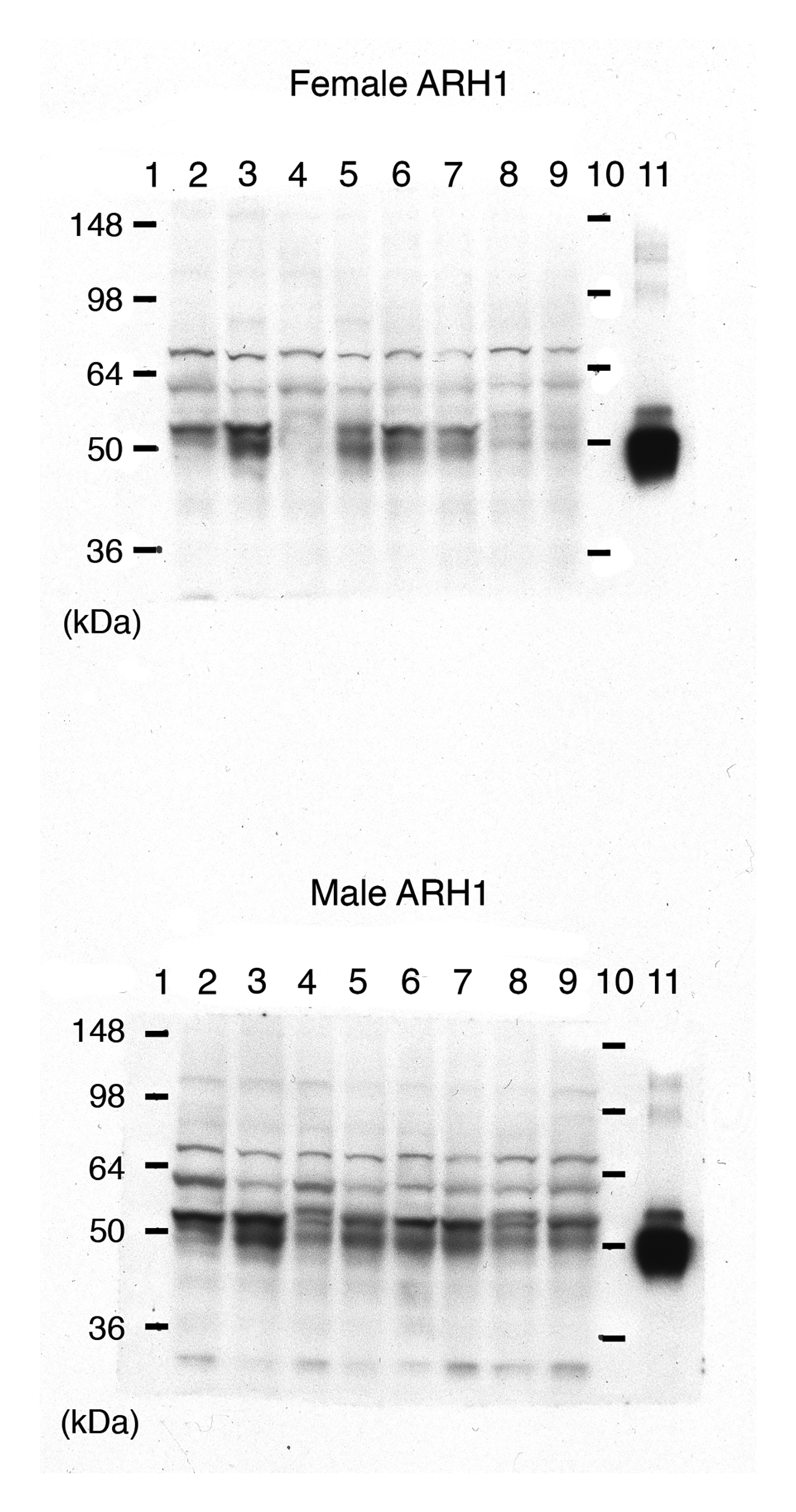

Supplement: S3 Fig — presents the original blots (raw data) of Fig 1C Gαs blots. Upper and Lower immunoblots using Gαs antibody show modified Gαs in intestinal loops treated with PBS, cholera toxin (CT) or 8-Bromo-cAMP (8-Br-cAMP) in female ARH1 KO and WT mice, and male ARH1 KO and WT mice, respectively. Lanes 2–7 in Western blots using anti-Gαs antibody was shown in Fig 1C. Lanes 1 and 10 show the protein molecular weight (kDa) using See Blue Plus Protein marker (Invitrogen, CA). Lane 11: positive control, recombinant Gαs protein (50 ng) (Millipore Sigma, MA). Lanes 2–7; Intestinal loops in female or male ARH1 WT and KO mice were treated with PBS, PBS containing 0.5 μg cholera toxin (CT) or 5 mM 8-Br-cAMP for 6 hours. Lane 2: ARH1 KO intestinal loops treated with PBS, Lane 3: ARH1 WT intestinal loops treated with PBS, Lane 4: ARH1 KO intestinal loops treated with CT, Lane 5: ARH1 WT intestinal loops treated with CT, Lane 6: ARH1 KO intestinal loops treated with 5 mM 8-Br-cAMP, Lane 7: ARH1 WT intestinal loops treated with 5 mM 8-Br-cAMP. In upper blot of female ARH1 KO mice; Lane 8: CT-treated intestinal loops for 2 hours in ARH1 KO mice, Lane 9: CT-treated intestinal loops for 4 hours in ARH1 KO. In lower blot of male ARH1 KO mice; Lane 8: CT-treated intestinal loops for 4 hours in ARH1 KO mice, Lane 9: CT-treated intestinal loops for 2 hours in ARH1 KO. (TIF) [file pone.0207693.s003.tif]
